# A Text-Book of Bacteriology

**Published:** 1897-06

**Authors:** 


**A Text-Book of Bacteriology.** By EDGAR M. CROOKSHANK, M.B. Fourth Edition. Pp. xxx., 715. London: H. K. Lewis. 1896.

The author of this well-known text-book claims in his preface to have produced "practically speaking a new work;" and a careful comparison with earlier editions will show that this claim is hardly exaggerated. An immense improvement has been effected by the re-arrangement of the matter, and by the incorporation of much new information, in which latter respect the work is well up to date. The removal of the somewhat scrappy accounts of comparatively unimportant species of organisms to an alphabetical appendix, and the systematic description in the body of the work of such forms as have been most thoroughly studied, is a change which, if it increases the similarity to other books covering the same ground, is of unquestionable advantage to the reader. It is perhaps a pity that the descriptions of apparatus are not all collected under one head; but as this is a section very commonly skipped by the student, little harm is probably done. It will appear to many that a rather disproportionate amount of space has been allotted, not perhaps unnaturally, to certain branches to which the author has devoted special attention, notably actinomycosis and smallpox. The latter disease and its relation to cowpox are treated at considerable length, in addition to which there is a copious extract from the report of the Royal Commission on Vaccination: though this matter can hardly be considered irrelevant, it seems a little out of place, considering its debatable character, and the very limited amount of bacteriological information we possess in connection with it. The illustrations continue to be a prominent and successful feature, the best of those which appeared in the earlier editions being retained, and reinforced by the addition of numerous new photographs and wood-cuts. The author may fairly be congratulated on the success that has attended the re-casting of this work, and with a little more judicious pruning of controversial matter the next edition will take front rank amongst text-books on the subject.

